# Foreign Body Removed from the Left Bronchus

**Published:** 1863-11

**Authors:** J. F. Miner


ART. III.—*Foreign body removed from the left Bronchus.*

BY J. F. MINER, M. D.

Mrs. T——, a patient in the Buffalo General Hospital, while eating dinner became very suddenly strangulated, and appeared for a time, as though she would never breathe again; at length, however, she was able to respire more easily, and finally became comparatively comfortable. When able to converse she said that her mouth contained, when she commenced dinner, two pins, that one of them was gone, and must have passed down her throat while eating her soup. The respiration becoming gradually more easy and the cough less severe than at first. It was hoped that the foreign body was not in the air passages, or if so, that it would be ejected, or was so situated as to remain harmless. On the sixth day after the accident I was invited to see and take charge of the case. Upon examination it was found that the whole left lung was obstructed, the air entering slowly and escaping in the same manner with extensive rhonchus. The seat of the obstruction could be easily and satisfactorily determined by the great amount of rale present at that point, and the impulse communicated by the forced respiration, The patient was now examined by many of the hospital staff, and also by several physicians not connected with the hospital. That there was some obstruction in the bronchial tube of the left lung was undoubted by all; that it was so complete as to seriously endanger life was also unanimously conceded, the irritation and inflammation together with the cough and disphnœa being so great that life could not be prolonged for many hours unless relief was afforded. In regard to the chances of success in attempting the removal, there was some differences of opinion, yet all agreed that they were few and uncertain; that to reach the seat of obstruction and remove a foreign body which appeared stationary and immovable, was nearly a hopeless undertaking. Some advised trusting to the chances of spontaneous elimination, and related cases of recovery by such method, but this case was certain to soon terminate fatally unless the foreign substance could be removed. It was also agreed that though it was not very probable it could be removed, yet an attempt was justifiable.

The patient was brought slowly and fully under the influence of sulphuric ether, and an opening made in the trachea as low as it was possible through the deep tissues of a fleshy young lady. After all hemorrhage had ceased, careful exploration was first made with a long silver probe, and the foreign body found distinctly present in the left bronchial tube, an inch or an inch and a half from the bifercation of the trachea. With small, long forceps, which had been bent for the purpose, an attempt was now made to seize and remove the offending substance, whatever it might prove to be. This was the difficult part of the operation, for respiration was frightfully embarrassed while yet the trachea was left unobstructed; any additional interference could be tolerated but for a few seconds only. After a few unsuccessful attempts our efforts were crowned by complete success, the foreign body being grasped and removed entire. It proved to be a piece of bone from the soup she was eating at the time of the accident. It was a little over an inch in length by half an inch in width, flattened a little, pointed and roughened. It had been inhaled and fortunately deposited in position where respiration could be continued by the opposite lung. The wound healed kindly, and all the symptoms of pulmonary irritation gradually subsided so that the patient was dismissed, the tenth day after the operation.

After the inhalation of foreign substances into the air passages, there are a very small proportion of cases of spontaneous ejection during violent paroxysms of coughing; these are usually instances where the foreign body is small and smooth, and is moving up and down in the trachea. Upon this ground a somewhat distinguished surgeon from Boston who was visiting the hospital at the time, and other medical men who were present advised trusting to the chances of spontaneous elimination, and related cases where kernels of corn, beans, and similar articles had been expelled. The hospital staff was united in the opinion that an effort to remove it was fully justifiable.

This advice, to wait the natural issues, has an appearance of conservatism about it. Conservatism in surgery does not consist in delay in making necessary operation, or in trusting to natural agencies for relief because nature may have been known to have put forth almost miraculous effort, and in rare instances succeeded. Conservatism consists in discrimination of cases, with energy and ability to plan and execute whatever may be necessary for the accomplishment of desired objects. This substance did not move, it was stationary, and there was no ground of expectation that it could ever spontaneously make its escape. It was impacted firmly in the tube, and no natural effort could cause it to change locations. It was producing great distress, and had remained so long that there was considerable purulent secretion. The bone itself was so roughened and of such shape, that when passed within the tube nothing could be supposed capable of extracting it, except the forceps. That foreign substances lodged in the air passages have been known to make their exit at other and distant parts of the body, having formed channels of exit by slow and gradual ulceration, the passage way being closed behind by the wonderful provisions of nature to protect and repair herself, forms no good ground of expectation that such results will follow sufficiently often to influence the plan of practice.

It may be said that after the successful removal, it is safer to talk of the propriety of such operation than before, while the character and shape of the foreign body was uncertain and the possibility of reaching it yet undemonstrated. If the effort had not been crowned by success; if the patient had died in the attempt, the satisfaction afforded by the fact that vigorous effort was made to save life, would have compensated in great degree, for the chagrin and mortification of failure, and would have been much more creditable to the energy and skill of those having charge, than to have neglected such effort.

